# Three-Dimensional Urethral Profilometry—A Global Urethral Pressure Assessment Method

**DOI:** 10.3390/diagnostics11040687

**Published:** 2021-04-12

**Authors:** Wioletta Katarzyna Szepieniec, Hanna Szweda, Maksym Wróblewski, Paweł Szymanowski

**Affiliations:** Department of Gynecology and Obstetrics, Faculty of Medicine and Health Sciences, Andrzej Frycz Modrzewski Krakow University, 30-705 Krakow, Poland; k.szepieniec@interia.pl (W.K.S.); msw1810@o2.pl (M.W.); pszymanowski@afm.edu.pl (P.S.)

**Keywords:** urodynamics, urethral profile, urethral pressure, assessment method, profilometry

## Abstract

Background: To present a new method of urethral pressure examination, and to evaluate diagnostic capabilities of three-dimensional profilometry, as an alternative to classical urethral profile (UPP). Using five channel catheters and dedicated software, a global urethral pressure image is obtained. The method eliminates the main limitation of classical urethral profilometry, where the catheter orientation determines the pressure picture limited to only one point in the urethral circumference; we observed up to 50% differences in pressure measures depending on the point of urethral circumference where the measurement was taken. Methods: This is a preliminary study containing a method presentation and analysis of the use in varied clinical cases of either healthy patients or patients with lower urinary tract symptoms (LUTS). The article includes a technique and equipment description and a full evaluation of selected cases, including three-dimensional urethral pressure distribution graphics. Results and Conclusions: Three-dimensional profilometry compared to the classical technique is comparable regarding the time, cost, technical difficulty and patient discomfort. At the same time, we obtained much more data on the urethral pressure and its distribution. The results are easy to interpret due to the 3D movable graphics created automatically by the dedicated software.

## 1. Introduction

In use since the 1970s, urethral profilometry (UPP—Urethral Pressure Profile) is one of several measurements utilized as part of a full urodynamic diagnosis. It does, however, have a number of limitations as a test in everyday clinical practice. One of these disadvantages is its low reproducibility. This limitation results from the use of traditional catheters, which measure urethral pressure only from one direction of the urethral circumference. The anatomical structure of the urethra means that the dimensional pressure distribution is very different depending on the direction from which the measurement is taken. In patients who have a pathology of the lower urinary tract, or who have had surgery or injuries in this area, this variation in pressure distribution is impaired.

The study presented in this paper allows for the assessment of urethral pressure (Pura) along the entire length of the urethra. Pressure readings are taken at the same time in four directions radially every 90° with simultaneous measurement of the intra-bladder pressure (Pves).

Three-dimensional profilometry allows for more precise and global assessments of the peripheral pressure distribution in the urethra. This type of measurement results in the elimination of the effects of the catheter being rotated or imprecisely placed during examination which can very often produce false readings. By using the three-dimensional profilometry, the imperfections of classical urethral profilometry are reduced or even completely eliminated.

Urinary incontinence or involuntary urination, which is so prevalent in the world that WHO qualifies it as a social disease, can occur at any age, but it is most common among the elderly population, twice more common in women than in men [1].

In the majority of patients, however, this chronic symptom can be completely cured or reduced after a proper diagnosis. According to the literature, the vast majority of patients with incontinence can achieve significant improvement with a well-chosen treatment. Referring to stress urinary incontinence (SUI) up to 90% of patients [2] and in case of mixed urinary incontinence (MUI) and urge urinary incontinence (UUI) in over 60% of patients, UI symptoms are either reduced or eliminated [3,4].

As many as 50% of women between the ages of 45 and 60 have experienced an incontinence incident [5] One of the main risk factors of urinary incontinence is childbirth (stress urinary incontinence is diagnosed in up to 30% of women after physiological delivery and up to 31% after delivery by forceps) [6].

A common method of assessing the function of the urethra in stress urinary incontinence is profilometry, although this method is often criticized for its poor repeatability [7].

Current methods used to measure urethral pressure utilize different types of catheters Regardless of the type, the measurement of urethral pressure with one channel catheter does not give the opportunity to detect any asymmetries in pressure distribution [8].

Catheters recommended for measurement of urethral pressure by the International Continence Society (ICS) are water-perfused systems. These are considered to be the most accurate and allow researchers to obtain reproducible and comparable results in contrast to micro-tip or air-filled catheter systems [9,10]. At the same time, ideal methods for differentiating continent patients from stress incontinent are still searched, and there is also a lack of tools for assessing the treatment methods based on pathophysiological changes in the urethra.

The urethra is not a symmetrical, thick tube. The pressure at different points of its circumference differ significantly [11,12], which results from the urethra and pelvic floor anatomy and function. The urethral sphincter has a horseshoe shape opened dorsally. Moreover, the shape, functional length, stiffness, and mobility of the urethra are defined by other pelvic floor structures surrounding the urethra on different levels. A classical urethral profile measurement is taken along the urethra, but from only one point of urethral circumference.

In view of these shortcomings when using traditional methods to measure the urethral pressure, it is desirable to perform measurements over the entire length and whole circumference of the urethra. An advantage of these measurements over their classical counterparts is their reproducibility and independence from examination, e.g., the cough test [13,14].

The purpose of this pilot study using the system described below is to evaluate a method of measuring urethral pressure changes more accurately, regardless of the peripheral position of the sensors in the urethra. Three-dimensional profilometry allows to create picture of the pressure distribution along the whole urethra and around its whole circumference. The conception is based on the methodology of anal sphincter manometry, extrapolated on urogenital tract conditions. In the 1990s, there were some in vitro trials of this kind of urethral pressure assessment [9] and attempts to use the method in animals appeared promising [15,16]. Regarding these data, in our work we have skipped the biophysical and in vitro testing stage. We used the idea of anal sphincter manometry, which has been used in clinical practice for years [12,15,16]. Seeing its potential in clinical practice, we performed, as a pilot study, the examination in women. A further purpose of this work is to present a three-dimensional approximation of pressure distributions in the urethra in order to better understand the mechanisms of incontinence. The technique of either classic and three-dimensional profilometry requires the use of a special catheter and puller, which is an integral part of urodynamic devices. The catheters used do not differ from the standard stiffness but are slightly thicker than the most commonly used catheters (9 Fr vs 8 Fr; catheter sizes of up to 10 Fr are allowed according to ICS recommendations) and the examination technique is the same as in the classic urethral profilometry. In addition to 3D profilometry, we use special software which allows to create movable three-dimensional reconstruction of the urethra. The time of the assessment and its complexity do not differ from the classical method. It takes less than 10 min, which considering the whole complex urodynamic study seems to be a short time added. The main difference is the number of sensors used and, hence, the amount of data collected. Thus, it can be assumed that the idea of the test, with respect to patient discomfort, tolerance, or duration of the examination, will not differ from those when using the classical method. Thus, using the 5-channel catheter and dedicated software, the performance does not differ for the investigator, nor for the patient. However, the amount of data collected during the three-dimensional profilometry is significantly greater when compared to the conventional method [15,17,18]. Regarding the reproducibility of three-dimensional UPP, we already started the reproducibility trial as the next stage of our studies on the method. However, reports from 1997 are promising and showed high reproducibility of multichannel profile measurements [15,18]. Further research on a larger group of patients is also needed to describe the characteristics of three-dimensional profilometry for specific pathologies. We continue the research fulfilling these expectations.

## 2. Materials and Methods

### 2.1. Settings and Participants

The research was carried out in the Department of Gynecology and Urogynecology at the Andrzej Frycz Modrzewski Krakow University from June 2018 to October 2018. The measurements were performed on a group of 25 women presented in the clinic for the treatment of urinary incontinence or after previous pelvic floor repair surgery or mid-urethral tape operations. The study included symptomatic as well as asymptomatic patients. The control group consisted of patients who did not report problems related to urinary incontinence or other disorders within the pelvic floor (four patients). All patients were at least 18 years old, not pregnant and showed no significant prolapse of the pelvic organs of more than POP Q 1. The patients in the control group, without any symptoms of urinary incontinence, showed no signs of SUI either in the questionnaire or in the cough test, and had not previously undergone any surgery due to urinary incontinence or pelvic floor repair. The patients in the incontinence group were patients either with clinical symptoms during examination or with symptoms of mixed incontinence or an overactive bladder. Asymptomatic as well as symptomatic patients after surgery due to SUI or pelvic floor repair were also included in this group. The questionnaire presented to patients included the following information: age, height, weight, BMI, gynecological operations, parity, types of childbirth, weight of the largest child, whether they smoked and family history of urinary incontinence. They filled out a short form for Incontinence Severity Index (ISI, scale 0–12). The patients who took part in the study were volunteers and were not associated with the authors in any way.

Measurement: The examination was conducted according to the ICS standards [10].

Urethral pressure measurements: After micturition, a Foley catheter 12 or 14 Fr is used to empty the bladder of residual urine. Next, using the catheter, the bladder is filled with 200 mL of sterile water at room temperature or slightly warmer. In patients with reduced bladder capacity, the bladder is filled to the maximum volume, before it causes discomfort for the patient. After removal of the catheter, a cough test is performed to confirm the absence or presence of leakage of urine. While the patient is in a semi-sitting position, the catheter is inserted into the urethra to a depth guaranteeing that all the sensors are in the bladder. In order to confirm that the catheter has been accurately placed, a cough test is again performed. The auxiliary line on the catheter (indicating channel Pura1) should be directed ventrally. The catheter is then attached to a pulling mechanism, which is used to pull the catheter out. The speed of the catheter withdrawal in each case was 1 mm/s. The resting urethral profile is performed twice on each patient and the stress (dynamic) profile is performed once.

System for urodynamics PICO SMART: Measurements collected from the tests were processed using a computerized system for urodynamics, PICO SMART, software PICO3000 version 6.11., manufactured by MEDICA S.p.A., MODENA, ITALY.

The system was supplied with pressure modules SAU-LG (sampling rate—100 Hz per channel; measuring range from −100 up to +540 cmH2O; sensitivity: 0.15 cmH2O; accuracy 1% of full scale) with five independent pressure transducers MX960P1.

The profile was performed with a five-way water perfused catheter (5PPV-9) which was custom designed and manufactured by MEDICA S.p.A., MODENA—ITALY. The catheter was fitted with a central lumen, which was 1.1 mm in diameter with an opening at the tip for measurement of vesical pressure “Pves” and four lumens with four side-port openings of 0.8 mm in diameter, 6.0 cm apart from the central lumen. The side ports were radially arranged every 90° to measure urethral pressure “Pura” simultaneously from four different directions. The external diameter of the catheter was 9 Fr (3 mm), with a cannula length of 400 mm, and it was made of medical grade PVC. 

**Figure 1:**
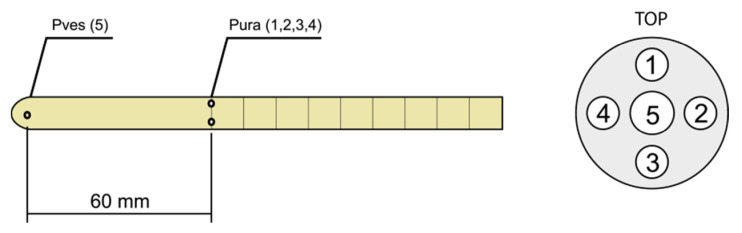
Placement of lumens in catheter 5PPV-9 (P ves- intravesical pressure, Pura-intraurethral pressure, 1–4 -channels numbers).

The catheter was equipped with five extension lines 180 cm in length, which connect the catheter to pressure transducers. (Figure 1).

**Figure 2:**
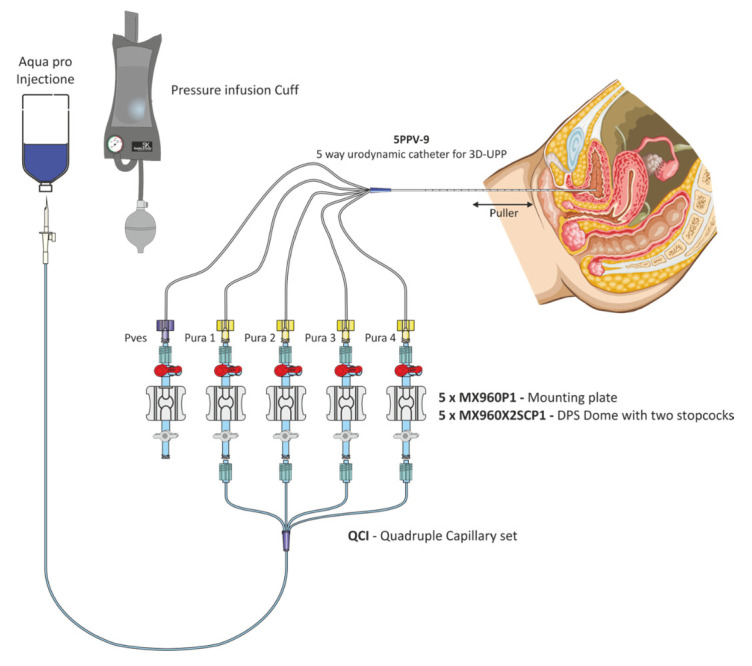
Connection diagram.

In order to achieve normal perfusion of the urethral channels while the measurements were taken, the Quadruple Capillary set (QCI), powered by a cuff pump, was used, and equal perfusion of 1 mL/min for all urethral channels was obtained because of this capillary set. Catheters configuration is presented on Figure 2.

Static UPP with 3D presentation.

Analysis of the static UPP is performed on each graph of Pura pressure. 

Static UPP automatically places the following markers:

F1: marks the onset of the profile (start of the high-pressure zone, beginning of the functional length).

F2: marks the top of the curve (Pura max).

F3: marks the end of the profile curve where the pressure returns to bladder values. It marks the point in which the catheter has passed through the striated sphincter. It is at this point that the profile functional length is calculated as the length of the urethral section where the pressure is higher than in the bladder.

The static UPP report shows the following details for each measured channel (direction):

- **Ev**: the number of profiles being measured.
- **P_ves**: pressure in the bladder.
- **P_ura max**: maximum urethral pressure measured in relation to marker F2
- **P_ucp**: maximum urethral closure pressure defined as P_ura max -P_ves
- **P-ave**: average urethral pressure
- **L_pmax**: length of maximum pressure; in other words, the distance in mm between the maximum pressure point and the start of the high-pressure zone (F2–F1).
- **L_fun**: functional length; in other words, the length of the section where the pressure is higher than the bladder pressure (F3–F1).
- **Area**: continence area. This is the area under the profile curve from marker F1 to marker F2. Dimensional analysis results in the following:
  [Pressure]*[Length]= MT^−2^ (1)
  mass divided by time squared.
  [Energy]/[Surface] = MT^−2^ (2)
  continence area has dimensions similar to energy per surface unit and represents the potential energy associated to the urethral sphincter strictly related with the urethral competence. The meaning of this parameter has not yet been fully explained.
- **Vector volume**: continence volume calculated from areas along the functional length of the urethra.
- **TLS**: total length of the sphincter.

### 2.2. Vector Volume

**Figure 3:**
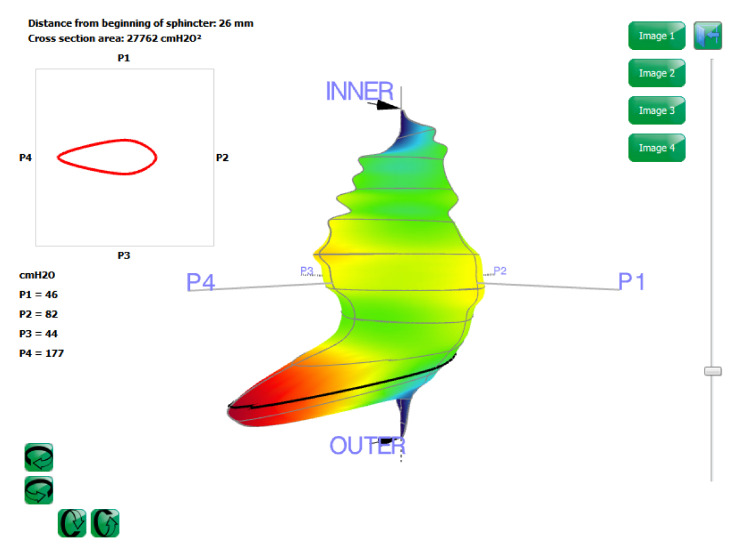
Cross section of the vector volume in correspondence with the distance from the beginning of the sphincter.

The analysis window presents a three-dimensional representation of the vector volume, shown in Figure 3.

The area is calculated by the following formula:

Area = 0.5[(P1 × P2)+…….(Pn-1 × Pn) + (Pn × P1)]sin [in cmH2O^2^] (3)

This represents the area of the polygon whose vertexes are (P1,….Pn). The angle between the vertices is 2π/n.

The scrolling bar on the graph allows the black vertical cursor to be moved, thereby changing the distance from the beginning of the sphincter. The pressures shown on the 3D graph and area values correspond with the position of the cursor.

One of the functions of the software is that the picture can be rotated by the scrolling buttons on the lower left side and screenshots can be taken by using the image buttons. These can then be inserted into the medical report. 

### 2.3. Dynamic UPP

The analysis of the dynamic UPP is performed on a selected graph showing the Pura pressure. It can be performed separately for each direction. The dynamic definer has the following markers:

Pves channel: 

F1: at the foot of each peak.

F2: corresponding to the Pves peaks

Pura channel:

F1: at the foot of each peak

F2: at the top of the Pura peaks

The program computes a data report with the numerical values of P_ura measured in correspondence to markers F1. Furthermore, it shows the distance of every event (peak) from marker “d” and the value of the transmission coefficient at every peak, defined in the following way:

TC% = 100(F2–F1 at Pura)/(F2–F1 at Pves)(4)

The urethral pressure profile is a test for the evaluation of incontinence. As an indication, the normal value of urethral closure pressure can be derived by the formula 110-age, in which values less than 20 or 25 cmH2O are surely pathological. For dynamic UPP, values of TC less than 80 are considered pathological.

## 3. Results

Below, we present examples of the three-dimensional profilometry evaluations according to above mentioned scheme.

Patient: G-001; Age (at examination): 28 years 

Examination date: 18 April 2018 

Prot. A—UPP S VETTORE; Puller: 1.0 mm/s

History: healthy control group patient without incontinence.

**Figure 4:**
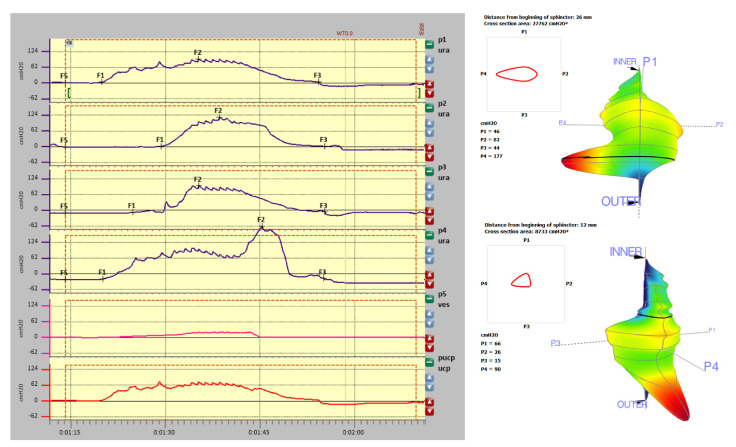
Resting profilometry graphic representation (left side: indyvidual channels measurements, right side-three dimensional reconstruction).

**Figure 5:**
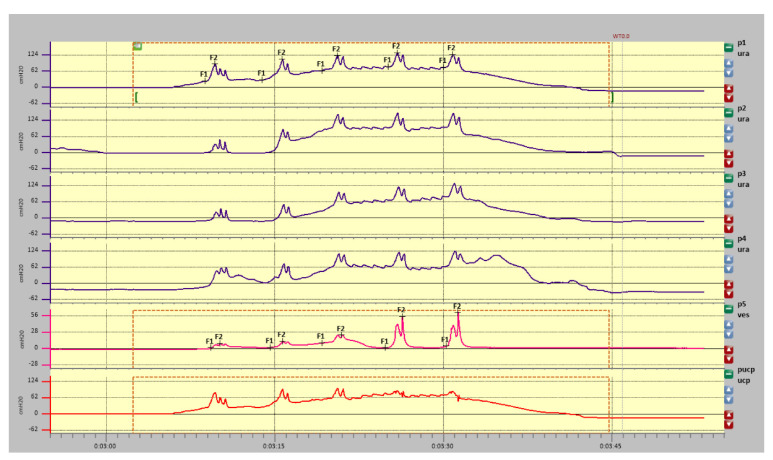
Stress profilometry.

Measurements are presented in Figure 4 (graphic representation of resting profilometry), Table 1 (measurements data), Figure 5 (graphic representation of stress profilometry) and Table 2 (stress profilometry measurements).

### 3.1. Resting Profilometry

There were significant differences in the functional length of the urethra (upper right side—3.5 cm, bottom left side—2.6 cm). Pura max was around 100 cmH2O, except for a peak increase at a distance of 26 mm from the internal sphincter on the right. This increase may suggest compression (obstacle) in this region. In the middle region of the urethra, uniform pressure distribution in the range of 80 cmH2O is observed. The maximal closure pressure of the urethra in all directions above 94 cmH2O is higher than the values typical for the patient’s age (110—age (28) = 82 cmH2O). What draws attention in this profile is a high difference between Pura max values in different channels—92.4 cm H2O between p4 and p 1, which is 50.5% in an asymptomatic patient.

### 3.2. Stress Profilometry

In provocation tests, positive closure pressures have been demonstrated over the entire length of the urethra. However, these values caused by provocative tests (cough pulses) do not exceed 40% of the average static closure pressure of the urethra. For a proper evaluation of the stress test (dynamic), it is recommended that provocative tests (cough impulses) reach about 80% of the average static closure pressure of the urethra. A high transmission coefficient TC (262.57%) is much higher than the value below, which possibly represents the presence of pathological changes.

Patient: G-006; Age (at examination): 51 years

History: patient with symptoms of stress urinary incontinence qualified for operation.

**Figure 6:**
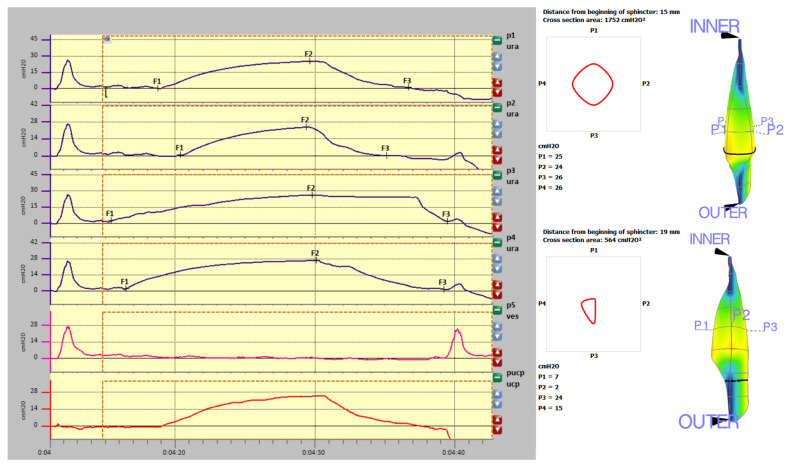
Rest profilometry.

Rest profilometry data is presented in Figure 6 (graphic presentation) and Table 3. 

### 3.3. Rest Profilometry

Significant differences in the functional length of the urethra (top and right side—1.8 cm, bottom and left side—2.4 cm). Pura max is around 26 cmH2O. In the central region of the urethra, the pressure distribution is equable at around 20 cmH2O. At a distance of 19 mm from the internal sphincter, urethra functionality is preserved only from the direction Pura3 (bottom); in all other directions, a rapid decrease in pressure readings is observed. The maximal closure pressure of the urethra in all directions below 26 cmH2O does not reach the values typical for the patient’s age (110—age (51) = 59 cmH2O).

### 3.4. Stress Profilometry

**Figure 7:**
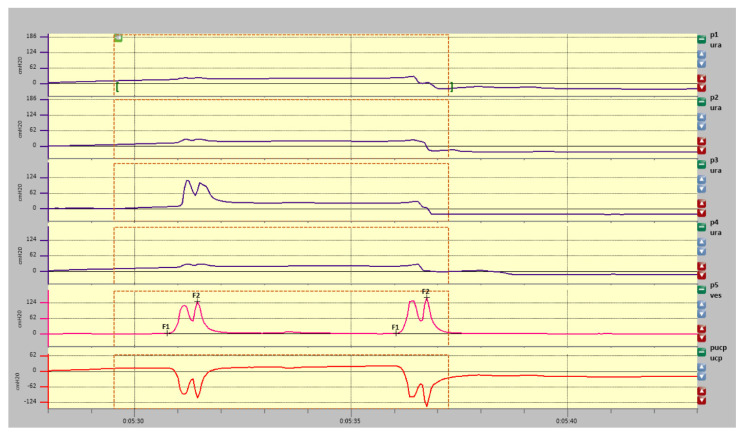
Stress profilometry.

The patient failed to perform proper stress profilometry. Any provocation test triggered immediate leakage of urine. A provocative test that increases the pressure inside the bladder to the value of 120–130 cmH2O did not increase the urethral pressure in the directions P1, P2 and P4; therefore, the analytical markers for these channels could not be located. Only in the P3 direction (rectum) at the very proximal part of the urethra was there an increase in pressure up to 72 cmH2O, but it was not sufficient to cause urethra closure. Closure pressure for this direction was negative (−57 cmH2O) (Figure 7).

The examinations which were performed using the dimensional profilometry method were well-tolerated by patients. The catheter, despite its specificity, did not cause discomfort to patients. 

The time needed to perform the tests was on average 2 min for tests at rest and 2 min for stress profilometry. The time necessary to conduct dimensional profilometry was not any longer than classical urethral profilometry.

## 4. Discussion

There are a few methods of urethral function assessment, and none of them are routinely used nor perfect. Urethral function is indirectly evaluated during filling and voiding cystometry (CLPP, VLPP). Imaging tests such as ultrasound or MRI examination allow to assess anatomical factors statically and dynamically (urethral length and mobility), and periurethral space pathologies. Direct functional tests and pressure rating is performed by static and dynamic urethral profilometry. In this test, apart from intraluminal pressures, the relation between vesical and urethral pressures is also rated [19]. Urethral profilometry, developed as a scientific tool, remains an additional test and is not routinely performed. However, in particular group of patients, it can provide useful and unique data, which, when combined with clinical data, is an important part of diagnostic and therapeutic process. Three-dimensional profilometry, with the same time, cost and complexity of the technique when compared with classical method, provides much more data, which is easy for interpretation for clinicians. It is even more useful for scientists working on a lower urinary tract function theme.

The present study resulted in the collection of detailed data on pressure distribution within the urethra. The presented method allowed to create three-dimensional pictures of urethral pressure, which is the main novelty of the assessment. Results are easy to interpret. Thanks to the ability to view and analyze the projection in movement, very detailed data are easy to obtain. 

In our project, we observed that the differences in pressure readings depending on the position of the catheter measurement channel in classic profilometry can be significant—even more than 50%. The 5PPV 9 catheter used in the method presented in this paper has 4 marked Pura measuring channels. Performing urethral profilometry four times using a classical catheter and changing the position of the measuring channel every 90 degrees would not get comparable results, due to changes in the muscle tonus of the pelvic floor and the tonus of the sphincter during subsequent tests. Simultaneous measurement at four points around the circumference of the urethra results in a significant advantage of dimensional profilometry over the traditional method. It was also discovered that in asymptomatic patients, there may be significant pressure differences in the urethra. 

It is interesting that depending on the location of the measurements taken regarding individual channels, differences in the functional length of the urethra were also found, with the P3 channel being the most variable indicator. In patients with stress urinary incontinence (eight patients in the database), three patients had the longest Lfun in channel P3, and in four patients, the shortest Lfun was in channel P3. The average difference between the longest and the shortest Lfun in these eight patients with SUI is 1.01 cm.

Another conclusion worthy of consideration applies to the analysis of test subjects who had undergone successful stress urinary incontinence surgery using a mid-urethral sling TOT. In an asymptomatic patient who was operated for SUI, we found negative pressure in the P1 channel over the entire length of the urethra. Such results confirm the fact that the effectiveness of a mid-urethral sling does not require the correction of pressure over the whole circumference of the urethra.

In patients who were examined with stress urinary incontinence, there is no clear tendency to maximum and minimum pressure values in specific directions of measurement.

A valuable parameter obtained during exercise profilometry is the so-called Transmission Coefficient (TC), which illustrates the transmission of pressure during provocative tests. It seems that the lower the TC, the higher the risk of pathological changes of stress urinary incontinence. This is clearly visible in patients with SUI and low pressure in the urethra, where the TC amounted to only 25%. We found out that TC is also lower in patients with initially high resting urethral pressure, which may indicate the impact of excessive tension of the pelvic floor on the function of the urethral sphincter. Higher values of TC correlate with positive SUI treatment effects.

A low TC (transmission coefficient) seems to be characteristic among patients with complaints of SUI, whereas in healthy patients, there is a significant variation in the value of this coefficient (30–267%). A potential subject of further studies requiring long-term observation would be to use the coefficient as a predictor of future incontinence in patients with low values of this parameter.

In the control group of patients without urinary incontinence, a significant variation in pressure distribution was found at each radially localized point of measurement. Differences were also found in the functional length depending on the orientation of the sensor—it is not known whether this has an impact on the frequency or severity of complaints. There were also significant differences in transmission coefficient values. A correlation was noticed between the initial intraurethral pressure in the static profile and the amount of pressure generated during provocation tests. This relationship, as well as its effect on the occurrence and type of symptoms and the causes of increased static pressure in the urethra, requires further investigation. The next parameter to be taken into consideration is L_pmax: (length at maximum pressure; in other words, the distance in mm between the maximum pressure point and the start of the high-pressure zone) it seems that in patients with stress urinary incontinence, there is a tendency for this point to be shifted and the value of this parameter increases.

## 5. Conclusions

Three-dimensional profilometry likely results in the elimination of testing errors due to changes in the position of the catheter in the measurement channel. By using three-dimensional profilometry, the dimensional distribution of pressure in the urethra can also be comprehensively assessed during a stress test. Three-dimensional pressure distribution images can be obtained using dedicated software, and these images do not require complicated analysis. The results of the preliminary studies show a significant variation in urethral parameters in the group of patients without symptoms. Thus, in the group of patients with incontinence of various types tendencies of certain characteristic, changes can be observed.

Establishing norms and characteristic values for different types of problems requires research on a larger group of patients. Trying to evaluate risk factors for urinary incontinence using specific parameters in healthy patients requires long-term observation. Examinations performed on patients who have already undergone urogynecological procedures could potentially lead to finding unknown risk factors, or uncovering factors which could determine the success of operations used to treat incontinence. It seems that this method, with a likely high reproducibility, allows for the collection of very accurate data on pressure distribution in the urethra. Dimensional images obtained using this method may lead to the rapid assessment of urethral dysfunction and also pinpoint its location, which may be useful in SUI surgery. It should be pointed out that this method will be useful in the diagnosis of the urethra, especially in the diagnosis of voiding dysfunction, complicated cases of urinary incontinence and periurethral space pathologies such as urethral diverticula. It can also be useful in the evaluation of urethral disorders in patients who have previously undergone procedures in the genital and lower urinary tracts, such as removal of an obstacle blocking the flow of urine in the urethra. Both the assessment of the repeatability of the study and the suitability for the assessment in specific clinical cases require further research on a larger group of patients. These further trials are already being performed in our department, together with 3D profilometry repeatability study.

## Figures and Tables

**Table 1:** Urethral pressures measurements data.

Ev	P_ves (cmH2O)	P_ura max (cmH2O)	P_ucp (cmH2O)	P_ave (cmH2O)	L_pmax (cm)	L_fun (cm)	Area (J/m^2)	VV (cmH2O ^2*mm)	TSL (cm)
p1 ura p2 ura p3 ura p4 ura	19.9 20.2 19.9 1.3	94.5 114.6 95.9 186.9	74.5 94.4 75.9 185.7	51.8 56.5 47.4 86.1	1.5 0.9 1.1 2.5	3.4 2.6 3.0 3.5	84.0 58.0 35.0 224.0	255,800.0	3.5

**Table 2:** Stress profilometry measurements data.

Profile	P_ura in F1	Pucp in F2	Distance from ‘d’	TC
	(cmH2O)	(cmH2O)	(cm)	(%)
Peak 1	63.1	98.6	1.86	601.11
Peak 2	77.7	76.7	2.4	100.28
Peak 3	75.3	63.5	2.89	86.3
Average transmission rate:				262.57

**Table 3:** Rest profilometry measurements data.

Ev.	P_ves (cmH2O)	P_ura max (cmH2O)	P_ucp (cmH2O)	P_ave (cmH2O)	L_pmax (cm)	L_fun (cm)	Area (J/m^2)	VV (cmH2O ^2*mm)	TSL (cm)
p1 ura p2 ura p3 ura p4 ura	2.8 2.7 2.7 2.4	25.4 23.8 26.3 26.5	22.6 21.1 23.6 24.1	15.4 14.5 17.5 17.5	1.0 0.8 1.4 1.3	1.6 1.2 2.3 2.0	17 13 21 25	11,514.7	2.3

## Data Availability

Data available on request due to restrictions (privacy, marketing issues). The data presented in this study are available on request from the corresponding author.
